# Umbilical Cord Blood Transplantation: Connecting Its Origin to Its Future

**DOI:** 10.1093/stcltm/szac086

**Published:** 2023-02-13

**Authors:** Gabriela Sanchez-Petitto, Katayoun Rezvani, May Daher, Hind Rafei, Partow Kebriaei, Elizabeth J Shpall, Amanda Olson

**Affiliations:** Department of Stem Cell Transplant and Cellular Therapy, The University of Texas M.D. Anderson Cancer Center, Houston, TX 77030, USA; Department of Stem Cell Transplant and Cellular Therapy, The University of Texas M.D. Anderson Cancer Center, Houston, TX 77030, USA; Department of Stem Cell Transplant and Cellular Therapy, The University of Texas M.D. Anderson Cancer Center, Houston, TX 77030, USA; Department of Stem Cell Transplant and Cellular Therapy, The University of Texas M.D. Anderson Cancer Center, Houston, TX 77030, USA; Department of Stem Cell Transplant and Cellular Therapy, The University of Texas M.D. Anderson Cancer Center, Houston, TX 77030, USA; Department of Stem Cell Transplant and Cellular Therapy, The University of Texas M.D. Anderson Cancer Center, Houston, TX 77030, USA; Department of Stem Cell Transplant and Cellular Therapy, The University of Texas M.D. Anderson Cancer Center, Houston, TX 77030, USA

**Keywords:** adult hematopoietic stem cells, stem/progenitor cell, stem cell transplantation, cord blood, umbilical cord blood

## Abstract

Transplantation of umbilical cord blood (UCB) is an attractive alternative source of hematopoietic stem cells (HSCs). The unique properties of cord blood and its distinct immune tolerance and engraftment kinetics compared to bone marrow (BM) and peripheral blood progenitor cells, permit a wider disparity in human leukocyte antigen levels between a cord blood donor and recipient after an unrelated umbilical cord blood transplant (UCBT). In addition, it is readily available and has a lowered risk of graft-versus-host disease (GvHD), with similar long-term clinical outcomes, compared to BM transplants. However, the relatively low number of cells administered by UCB units, as well as the associated delayed engraftment and immune reconstitution, pose limitations to the wide application of UCBT. Research into several aspects of UCBT has been evaluated, including the ex vivo expansion of cord blood HSCs and the process of fucosylation to enhance engraftment. Additionally, UCB has also been used in the treatment of several neurodegenerative and cardiovascular disorders with varying degrees of success. In this article, we will discuss the biology, clinical indications, and benefits of UCBT in pediatric and adult populations. We will also discuss future directions for the use of cord blood.

## Teaser

Umbilical cord blood stem cells are an alternative source of hematopoietic stem cells that allows greater disparity between HLA levels between donors and recipients. - Cord blood stem cell transplantation has comparable clinical outcomes and a lower risk of graft-versus-host disease than other sources of stem cells. - Limitations in terms of the low number of cells administered by units and slow engraftment, are areas in which additional research is being conducted. - Besides their application in oncology, they are also used in the treatment of several neurodegenerative and cardiovascular disorders

## Introduction

Umbilical cord blood transplant (UCBT) is a very effective alternative source of hematopoietic stem cells (HSCs), especially for patients with hematologic conditions where there are no available unrelated or related donors who are human leukocyte antigen (HLA) identical.^1^ Despite the fact that over 39 million donors are registered with the National Marrow Donor Program (NMDP) and affiliated registries, many patients, especially those from minority groups, will not be able to find a suitable, unrelated donor within the required timeframe.^2^ Umbilical cord blood (UCB) has expanded transplant eligibility for patients of racial and ethnic minorities across the US and worldwide.^2-4^

Since the first UCBT in 1988, over 40 000 UCBTs has been performed, and over half of the patients have been cured with this technique.^5^ In the last few decades, pediatric UCBT have been performed with high success rates both for hematologic conditions as well as metabolic storage diseases.^6,7^ Similarly, the success rates for adult UCBT have improved, after an increased emphasis on cord blood units that contain a sufficient cell dose, in addition to the use of double cord blood units in adults instead of a single cord, improved conditioning regimens, ex vivo expansion, fucosylation, among other strategies.^8^

UCBT has several advantages over bone marrow (BM) and peripheral blood progenitor cells (PBPCs) graft sources, including less stringent HLA-matching criteria, and the availability in both private and public health banks of cryopreserved specimens (Table 1).^9,10^ Furthermore, the naïve state of immune cells present in cord blood leads to a lower incidence of graft-versus-host disease (GvHD).^9^ The disadvantages are a slower engraftment of neutrophils, platelets, and overall immune recovery.

**Table 1.** Benefits and disadvantages associated with UCBT.

**Benefits**
High concentration of stem cells
Rapid availability with no risk to the donor
Low immunogenicity
Less HLA-match stringency
Increases donor pool
Decreased risk of chronic GvHD
Availability in both private and public health banks
**Disadvantages**
Low cell dose in single cord blood unit
One-time supply
Delayed engraftment and immune reconstitution
Risk of graft failure
Increased TRM
Increased risk of infections
High storage cost

Abbreviations: HLA, human leukocyte antigen; GvHD, Graft-versus-host disease; TRM, treatment-related mortality; UCBT, umbilical cord blood transplantation.

In the last few decades, many strides have been made to address the limitations of UCB with respect to delayed engraftment, including ex vivo expansion with coculture with mesenchymal cells, fucosylation of stem cells, among others. In this review, we examine the development of the field over the last several decades and its future prospects.

## Transplantation of Cord Blood: Scientific Rationale

Hematopoiesis normally occurs in intraembryonic (yolk sac) as well as extraembryonic sites (ventral aortic vessel) before relocating to the liver and BM. After birth, HSCs can be found almost exclusively in the BM as they circulate in the blood only during fetal development. The BM is the major source of hematopoiesis in adults.^11^

A growing body of research has focused on cord blood stem cells due to their proliferative advantages, including the capacity to produce autocrine growth factors, higher levels of proliferation potential, and longer telomeres.^12,13^ Research into fetal stem cells began in the 1980s after in utero transplantation of fetal HSCs revealed the hematopoietic activity and decreased immunogenicity of fetal liver cells as potential sources of stem cells.^14,15^ During initial experiments with sublethally irradiated mice with severe combined immunodeficient (SCID) treated with fractionated human cord blood cells, scientists found that high rates of long-lasting multi-lineage engraftment were achieved. Unlike adult human BM transplanted into mice, treatment with exogenous human growth factors was not necessary to facilitate engraftment.^16^

Later, HE Broxmeyer from the Indiana University School of Medicine and EA Boyse from Memorial Sloan Kettering (MSK) examined cord blood hematopoietic cell precursors for the purpose of enabling hematopoietic reconstitution in humans. Based on the analysis of 101 samples from New York and Indiana, they concluded that umbilical cords have granulocyte-macrophage colony-forming units (CFU-GM) in numbers comparable to those found in BM, a factor associated with successful transplantation. Furthermore, progenitor cells remained viable in unprocessed cord blood, and under low temperatures (4 °C), indicating that these cells could be successfully cryopreserved.^17^

The possibility of evaluating this approach for first time in Fanconi anemia (FA) patients was suggested by B Dupont from MSK and facilitated by AD Auerbach and H Friedman from the Rockefeller University and Duke University, respectively.^15^ Pregnant women with FA children were selected; the researcher used cultured amniotic fluid cells for the prenatal diagnosis and identified 5 mothers expecting a baby who was known, before birth, to be unaffected by FA and to carry the same HLA as the patient. UBC was harvested and cryopreserved at birth.^15^ As a result of this collaborative effort, the first UCBT was conducted.

## Viewpoint from the Past

In October 1988, the first UCBT was performed on a 5-year-old boy suffering from severe FA who did not have any available related or unrelated donors. His name was Matthew Farrow. As a result of the collaborative efforts of HE Broxmeyer and E Gluckman, he was the first patient to be treated with cryopreserved UCB from a non-affected, HLA-compatible sister identified by prenatal testing. The transplant was performed at the French Hôpital Saint-Louis in Paris. They collected Matthew’s sister’s cord blood cells at the time of her birth and proceeded with UCBT a few months later. Since FA patients are more susceptible to DNA cross-linkage and oxidative damage caused by alkylating agents, Matthew received cyclophosphamide and total body irradiation (TBI) as part of a reduced intensity conditioning (RIC) regimen. He developed grade I graft-versus-host disease (GvHD) of the skin in the immediate post-transplant period, which was successfully treated with intravenous steroids. He achieved neutrophil engraftment approximately 36 days following the transplant, and his counts continued to improve for several months.^18^ He is currently in his 30s and is currently cured of FA.

After the first UCBT, the concept was further expanded. In a phase I, study patients with leukemia, severe BM failure syndrome, and inborn metabolic errors were treated with unrelated HLA-matched or HLA-1 to 3 antigen disparate cord blood grafts obtained from the New York Blood Center (New York, NY). All patients following UCBT experienced successful engraftment with no late graft failures. Only 11% of the patients developed acute GvHD grades III–IV, and 65% of patients were alive at 6 months.^19^

As a result of this first successful transplant, UCB banks started to establish standard operating procedures for the collection and cryopreservation of UCB for related and unrelated uses. Pablo Rubinstein, under the direction of the New York Blood Center, established the first unrelated UCB bank in the US, with the largest cohort known at the time.^20^ The Cord Blood Transplantation Study (COBLT) Study Banking Program was initiated in 1996 with the objective of building a bank of unrelated cord blood donors to support transplantation protocols.^21^ Between 1998 and 2001, more than 17 000 cord blood units were collected. During the banking process, they found that higher UCB volumes and cell counts were obtained from cesarean sections compared to vaginal deliveries and that birth weight was correlated with both volume and cell content. Moreover, the study demonstrated that standard operating procedures and data collection can be established across multiple banks in a manner coordinated by 1 medical coordinating center.^21^ Currently the global Cord Blood Bank Inventory includes over 1 million cord blood units world-wide.

The phase II multicenter COBLT study was a National Heart, Lung and Blood institute-sponsored study, in which pediatric patients with lysosomal storage diseases, and adult and pediatric patients with hematologic cancers were included in 3 different cohorts to receive single UCBT.^6^ In the cohort of children with hematologic malignancies results compared favorably with those published from registry data with 1-year survival rate of 57%. The median time to neutrophil recovery was 27 days, while the median time to platelet engraftment was 174 days. The relapse rate was 19%; approximately 20% of patients developed acute GvHD of grades III–IV, and approximately 20% chronic GvHD.^6^ In a similar-aged cohort of 69 children with storage diseases, UCBT provided an overall survival (OS) rate of 72%, with a median time to neutrophil engraftment of 25 days. They observed a much lower incidence of chronic GvHD than would have been expected from unrelated marrow transplantation; the majority of patients had limited involvement without lasting consequences.^7^

Thirty-four adults with hematologic malignancies were included in the adult stratum. Unfortunately, the group’s survival was not as good as that of the pediatric group: approximately half of the subjects were dead at day 100, and only 17% were alive after 12 months. Poor-risk disease was considered a significant factor contributing to early mortality in these adults. Moreover, the median time to neutrophil and platelet engraftment was 42 and 180 days, respectively, grades II–IV GvHD developed in 34% of patients.^22^ The study recognized that, in comparison with unrelated donor BM, UCB has the advantage of rapid availability and lower risk of severe GvHD despite differences in donor-recipient HLA.^7,22^

## Donor Selection: Related Versus Unrelated UCBT

The Eurocord developed a study through which they gathered information approximately 143 UCBT procedures performed at 45 hospitals from 1988 to 1996. Comparing patients who received UCBT from related and unrelated donors, the 1-year survival for recipients of UCBT was 63% and 29%, and neutrophil engraftment occurred in 50% and 94%, respectively. Overall survival at 1 year was 73% for recipients of cord blood that was HLA-matched, compared to 33% for recipients of mismatched grafts for 1 or more HLA antigens. This did not affect OS in patients who received unrelated cord blood, possibly because the researchers did not utilize high-resolution molecular typing. The incidence of acute GvHD for recipients of cord blood from related donors rose with the number of HLA mismatches.^23^ Several subsequent studies evaluating UCBT from unrelated donors in patients receiving myeloablative conditioning (MAC) therapy revealed acceptable rates of engraftment and GvHD. Further, a low total nucleated cell (TNC) dosage or a low CD34+ cell dosage was associated with poor engraftment and survival. This was, therefore, identified as a potential pitfall with single-unit UCBT and that strategies are needed to improve its outcomes.^24,25^

## Improving the Efficacy of UCBT

### Double Cord Blood Transplant: Is it Better to Double?

As a result of the delayed engraftment associated with a single UCBT, researchers attempted to improve its efficiency by performing 2 cord blood transplants (double cord blood transplantation or dUCBT). However, this posed concerns regarding accelerating engraftment at the cost of an increased risk of GvHD owing to the addition of HLA mismatches. In a pilot study conducted at the University of Minnesota evaluating adults and children using myeloablative dUCBT, all patients engrafted at a median time of 23 days with a low incidence of severe acute GvHD in spite of HLA disparity. Sustained hematopoiesis was derived from a single graft (the one with the highest CD3+ cell count), which predominated as early as 3 weeks following transplantation. It was believed that this donor predominance was immune-mediated.^26^ Based on these promising results, researchers replicated the pilot study in a larger pediatric population. There was no significant difference in survival rates between recipients of dUCBT but there was an elevated incidence of severe acute GvHD, as well as extensive chronic GvHD after dUCBT. Researchers concluded that dUCBT did not provide a survival advantage to children and adolescents.^27^

Following this, a retrospective registry study from the CIBMTR, the National Cord Blood Program, and the New York Blood Center supported the efficacy of dUCBT in adults, in that it was associated with similar rates of neutrophil recovery, treatment-associated mortality, and survival as those observed in recipients of a single UCB unit with an adequate dose of 2 × 10^7^ TNC/kg.^28^ However, higher rates of acute grade II GvHD were seen after dUCBT in the period 2002–2004, which the authors attributed to the use of non-ATG-containing regimens.^28^ Further studies showed that among patients receiving single or double units the cumulative incidence of neutrophil engraftment did not differ, yet the majority of patients receiving dUCBT subsequently developed single graft chimerism, suggesting a booster effect from the “non-engrafting unit” which later seems to vanish. However, this observation was not corroborated in a large series of dUCBT from the Minnesota group.^29^ In addition, using dUCBT has been associated with a modest reduction in the risk of acute leukemia relapse, suggesting enhanced graft-versus-leukemia (GVL) activity with this modality.^30^

A study of single-unit dominance in humans was carried out in parallel with a study on murine models receiving dUCBT. While sustained hematopoiesis in humans resulted from a single unit of cord blood in all but one case, and similarly to the Minnesota group’s findings, single unit engraftment was associated with a greater number of CD3+ cells rather than progenitor cells, the unit predominance in mice was mediated by CD34− cells. Thus, it was concluded that unit dominance was considered to be an in vivo phenomenon mediated by CD34− cells as a graft-versus-graft immune interaction.^26,31^

### Conditioning Regimen in UCBT: To Ablate or Not to Ablate?

A number of studies have assessed the use of RIC regimens for UCBT despite initial concerns that the reduced alloreactivity of UCB may impair its ability to engraft following this regimen.^32^ A pilot study conducted by the Minnesota group evaluated donor engraftment following a dose-intensity de-escalation approach. Initially, the use of busulfan, fludarabine and total body irradiation (TBI) caused prolonged neutropenia; therefore, in an effort to achieve comparable immunosuppression with less myelosuppression, busulfan was substituted for cyclophosphamide. For GvHD prophylaxis cyclosporine (CSA) and mycophelonate mofetil (MMF) were used. Most patients benefited from the RIC regimen (cyclophosphamide, fludarabine, and TBI) in terms of rapid neutrophil recovery and complete donor engraftment.^32^

Another study used fludarabine, melphalan, and anti-thymocyte globulin (ATG) followed by 2 partially matched UCB units at a median TNC dose of 4.0 × 10^7^ cells/kg and 1.9 × 10^5^ CD34^+^ cells/kg in 21 adult patients. Similarly, CSA and MMF were used for GvHD prophylaxis. Treatment-related mortality (TRM) at 100 days was low (14%), as was the relapse rate, suggesting that the UCBT preserved the GVL effect over time. Noteworthy was that this was the first UCBT study to evaluate high-resolution HLA typing and matching between the 2 UBC units (For details regarding HLA typing, please refer to the section entitled “HLA Criteria: Progress made on HLA Typing”).^33^ Subsequently, the Minnesota group confirmed an increased incidence of grades II–IV acute GvHD after the transplantation of 2 partially HLA-matched UCB units compared with a single unit, and even when severe acute GvHD was present, the incidence of TRM was significantly lower following dUCBT. Risk factors for developing GvHD were the use of 2 UCB units, an RIC regimen, and the absence of ATG.^34^

### In the Absence of a Hla Match: UCBT versus Haploidentical HSCT

Concomitant with the most recent advances in GVHD prophylaxis, the use of haploidentical transplantation has increased over the years, similarly to UCBT, giving access to patients who do not have a match donor. A multicenter clinical trial randomized patients with hematologic malignancies to receive dUCBT or haploidentical HSCT: both groups received a RIC regimen. There were no differences in 2-year PFS between the donor sources, however patients had a shorter 2-year OS after dUCBT versus haploidentical transplantation and a greater non-relapse mortality (NRM). Neutrophil recovery after dUCBT was reduced, but the rate of GVHD was similar.^35^ A few limitations were highlighted in this study, such as the failure to complete accrual within 6 years of study initiation. Perhaps, this was indicative of the worldwide trend to use haploidentical donors rather than UCB grafts. Further, only the -DRB1 gene was analyzed at high resolution in the UCB units. Moreover, the authors used a RIC regimen instead of a MAC regimen, potentially affecting outcomes. We should point out that there was a higher crossover from the haploidentical group to the UCBT group, suggesting that UCBT may still have an important role whenever HLA differences exist.^35^

### Logistics and Financial Aspects of UCB Banking

In contrast to BM and peripheral blood collection, cord blood is collected from unpaid donors, processed, and banked.^36^ Worldwide, hundreds of thousands of cord blood units (CBU) are available in public and private cord blood banks for the treatment of multiple conditions.^37^ However, many financial limitations regarding the banking of UCB units exist. A report by the US Government Accountability Office indicates that cord blood banks face financial challenges in collecting more units at existing or new collection sites, as well as limited ability to cover the costs of hiring additional staff.^38^

Based on publicly available data, the Health Resources and Service Administration evaluated the number of UCBT and survival estimates for several diseases, and they concluded that the social benefits of cord blood banks of $500 million to $1.5 billion far outweigh the $60 to $70 million operating costs.^36^ Furthermore, a retrospective study comparing haploidentical HSCT with dUCBT, 2 modalities that allow less stringent HLA-matching criteria, and thus extending their access to ethnic minorities, found that haploidentical HSCT cost significantly less for graft acquisition and total charges.^39^ Obtaining haplo donor grafts is $53 000 less expensive than obtaining dUCB grafts. Furthermore, the dUCBT cohort had a numerically higher mortality rate (30% vs. 24%) which led to significantly higher total charges by an average of $101 000. As a result, every 20 haplo-HCT patients would save over $1 million in hospital charges. The authors suggested that in resource-limited circumstances, haploidentical HSCT can be an effective alternative to UCBT, although a prospective study is underway to clarify the clinical and financial benefits of these 2 transplant modalities (NCT 01597778).^39^

Nevertheless, these studies only consider a portion of cord blood transplantation and banking’s total social value, and they do not consider the future option value of having an integrated cord blood system in place for new or non-hematologic diseases as well as the possibility that technology will change the manner in which cord blood is used through expansion, fucosylation, and other methods.^36,39^

### The Quality of Life After UCBT

As the survival rate of cancer patients increases, there has been growing interest in the quality of life (QoL) of survivors.^40^ Despite this, there is a paucity of data comparing the QoL of long-term non-relapsed UCBT survivors with that of other transplant modalities. The survival outcomes of UCBT (without the use of ATG) and peripheral blood MUD transplantation, both using MAC regimens, were similar in a multicenter study conducted in China, but UCBT was associated with better QoL and lower incidence of chronic GvHD.^41^ To assess the quality of life of patients surviving for 2 years, Karnofsky performance score (KPS) was used. The post-transplant KPS of UCBT recipients was higher than that of unrelated peripheral blood HSCT recipients. Furthermore, chronic GvHD was lower in the UCBT group, which, according to the authors, may have contributed to the improvement in KPS. One of the limitations of this study is that was a retrospective analysis that did not use standard questionnaires for assessing the quality of life, but rather examined changes in KPS over time.^40^

An additional study assessed multiple measures of QoL after 1 year of HSCT, including functional assessment, fatigue, anxiety, depressive symptoms, suicidal thoughts, and sleep quality.^41^ UCB and MRD transplantation were compared in terms of QoL. Patients in the UCBT group were more likely to have longer hospital stays, receive more MAC regimens, experience less chronic GvHD, and take less immunosuppressants at the time of the survey. Despite not finding significant differences in the QoL outcome between the 2 groups, patients who take immunosuppressants (majority in the MRD group), a surrogate for chronic GvHD, scored lower on most surveys. In this study, low TERS scores (a tool used to assess the mental health and well-being of patients before transplantation) were independently and strongly correlated with post-transplant results: UCBT patients scored higher on TERS than MRD patients, and chronic GvHD patients scored lower.^41,42^ As a limitation of this study, a one-time survey was used rather than serial assessments at standardized intervals following transplantation. In assessing QoL among donor sources, the authors acknowledge that developing strategies to minimize chronic GvHD are likely to positively impact QoL.^41^

Based on their ability to return to work or school, long-term survivors who underwent UCBT had a better QoL as compared to those who underwent MRD transplants.^43^ A study showed that after 1 year of HSCT, 94% of UCBT patients were off immunosuppressants and returning to school or work full time. In contrast, 61% of MRD patients were still on immunosuppressants and only 60% returned to school or work. Once again, chronic GvHD posed the greatest threat to QoL, while stopping immunosuppressants and returning to work were crucially beneficial.^43^

### Choosing the Adequate Cord Blood Unit

In UCBT, optimal unit selection requires consideration of HLA-match, the quality of the unit, and the cell dose^44^.

### HLA Criteria: Progress Made on HLA Typing

Through registries, patients can find matched unrelated donors with a probability of 20%-70%.^45^ Thus, patients in need may benefit from haploidentical or UCBT, the latter offering both greater HLA mismatches without increasing GvHD risk.^45^ HLA incompatibility has long been recognized as a barrier to HSCT.^46^ Data relating HLA disparities to survival show a direct relationship between the number of donor–recipient mismatches and reduced survival.^47^ Due to the high degree of polymorphism of HLA molecules, older serological typing techniques (antigen-level resolution) have been replaced by the more recent reverse polymerase chain reaction (PCR) using sequence-specific primers, at an allele-level resolution.^48^

For BM and PBPCs transplants from unrelated donors, current standards recommend matching donors to the recipients at the allele-level for HLA-A, -B, -C, -DRB1, and -DPB1 loci.^44^ In a study of the CIMBTR and the European Group for Blood and Marrow Transplant (EBMT) allele-level matching between the UCB unit and its recipient was associated with improved survival and a reduced rate of graft failure. Furthermore, mismatching at HLA high-resolution types decreased neutrophil engraftment, increased graft failure, and increased mortality. These data support a shift away from conventional practices in the selection of UCB units for transplantation for non-malignant diseases that considers allele-level rather than antigen-level HLA matching.^49^Table 2 outlines the cord blood selection criteria outlined by the American Society for Blood and Marrow Transplantation (ASBMT) Cord Blood Interest Group.^44^

**Table 2.** Unrelated cord blood unit selection in accordance with expert recommendations.^44^

UCB cell dose^*^	
**Singleunit**	• Minimum cell dose: TNC ≥2.5 × 10^7^/kg and CD34^+^ cells ≥1.5 × 10^5^/kg. • Children with non-malignant diagnoses should receive higher cell doses (≥5 × 10^7^/kg). Note: Higher CD34+ dose is recommended in some centers.
**Double unit**	• Minimum cell dose: TNC ≥1.5 × 10^7^/kg (for each unit) *and* CD34^+^ cells ≥1.0 × 10^5^/kg (for each unit) Note: Higher CD34+ dose per unit is recommended in some centers.
HLA match	
**HLA-typing resolution**	• At least 8 high-resolution for both the patient and the cord blood unit, including HLA-A, -B, -C, and -DRB1
**Donor–recipient HLA-match**	• ≥4/6 HLA-A, -B antigen, -DRB1 by high-resolution and ≥4/8 by high-resolution match Note: Unit to unit HLA-match for dUCBT is not required.
**Other considerations**	
**Prioritization of cell dose vs HLA match**	• Children, smaller adults, and patients with common HLA typing who have multiple units with high cell dose: HLA-matching takes precedence. • Patients with non-malignant diagnoses: Optimizing HLA match is crucial. • High TNC and CD34+ cell dose: Optimizing the HLA matching over cell dose. • Lower TNC and CD34+ cell dose: Optimizing the cell dose first and then HLA matching. • Similar doses: Optimize high-resolution HLA matching.
**RBC-depleted units**	• Life-threatening reactions have been reported with the administration of RBC-replete units. • The use of these units should be limited to situations in which RBC-depleted cord blood units are not available.
**Significance of DSA**	• Non-malignant conditions: DSA targeted units should be avoided • Malignant conditions: DSA targeted units should be avoided if possible but use with caution in cases in which the avoidance of units against which the patient has antibodies compromises the chosen cord blood dose and/or HLA match.

^*^Cryopreserved units.

Abbreviations: DSA, donor specific antibodies; dUCBT, double umbilical cord transplant; GvHD, graft-versus-host disease; HLA, human leukocyte antigen; RBC, red blood cell, TNC, total nucleated cells; UCB, umbilical cord blood.

### Non-HLA Criteria: When Both Quality and Quantity Matter

Cord blood graft selection criteria must consider unit quality and cryopreserved cell dose, as well as HLA compatibility (Table 2).^50,51^ UCB unit quality is strongly related to unit potency, including post-thaw viability of CD34+ cells.^52^ Following the results of the Blood and Marrow Transplant Clinical Trial Network (BMT CTN 0501) trial comparison between single and dUCBT, an appropriately dosed single unit was defined as having a minimum TNC dose of ≥2.5 × 10^7^/kg.^27^ Whenever a single unit with a sufficient TNC dose is not available, which is not infrequent, 2 units are used, ideally containing a TNC dose of 1.5 × 10^7^/kg in each unit. In addition, CD34+ dose is now considered a standard measure in unit selection.^53^

In general, with UCB units, cell dose often needs to take precedence over HLA matching for adult patients, larger children, and those with nonmalignant diseases, whereas HLA-match can take priority in children or smaller adults and those with common HLA typing who have various high-dose units.^44^ Our current workflow for unit selection is as follows: if both the TNC and CD34+ doses are sufficient in all units, optimize high-resolution HLA match over the cell dose; and if the doses are low, optimize dose first and high-resolution HLA match second (Table 2).^44^

### Enhancing Engraftment and Administration of Stem Cells

#### Methods of Expansion

##### Ex Vivo Mesenchymal-Cell Coculture

The evidence available suggests that mesenchymal stromal cells provide important molecular signals required for the ex vivo expansion of stem cells; signals that are absent from conventional expansion methods based solely on suspension culture of hematopoietic progenitors in the presence of cytokines.^54^ In a study conducted by the MD Anderson Cancer Center (MDACC) group, ex vivo expansion of cord-blood cells was used to increase the number of myeloid and megakaryocyte progenitors. Thirty-one patients with hematologic cancers received MAC regimen and the infusion of 2 UCB units, of which one contained cord blood that had been expanded ex vivo in coculture with allogeneic STRO-3+ mesenchymal stromal cells. The results were compared to those of matched controls that received transplants of 2 units of unmanipulated cord blood from the MDACC and the CIBMTR.^55^

The coculture with mesenchymal stromal cells resulted in significant expansions of both TNC and CD34+ cells, as well as a shorter time to neutrophil engraftment (15 days in the expanded group vs. 24 days in the control group), platelet engraftment (42 days in the expanded group vs. 49 days in the control group). Despite being heavily pretreated and having the majority of patients with refractory disease at the time of entry, 35% of patients survived a 30-month median follow-up.^55^ Furthermore, the rate of GvHD was comparable to those in other trials of UCBT with and without expansion.^26,56^ The positive engraftment may be explained by the increased commitment of myeloid and megakaryocytic progenitors, which support early engraftment through the replication of the physiological properties of the stem-cell niche by mesenchymal cells. The presence of these factors enhances the survival and proliferation of cord blood progenitor cells. Nevertheless, as long-term engraftment was also obtained uniformly from cells from cord blood that had not been manipulated, it must also be admitted that the culture process appears to deplete the cells capable of long-term repopulation.^55^

##### UM171-Expanded Cord Blood

Human long-term-repopulating HSCs (LT-HSC) derived from human UCB have been proven to be capable of regenerating the lifelong production of all mature blood cells in murine models. UM171, a potent LT-HSC–stimulating agent, has been shown to expand stem cells and enhance multilineage blood cell reconstitution in mice.^57^ In a single-arm phase I/2 study 4 adult patients with hematologic malignancies received dUCBT (one expanded with UM171 and one unmanipulated) in the first phase, and in the second phase 22 patients received a single UM171-expanded cord blood unit with a dose de-escalation design to determine the minimal CBU cell dose to achieve early engraftment. After receiving a MAC regimen, patients were infused with the 7-day UM171-expanded CD34+ cells and the lymphocyte containing CD34− fraction. Neutrophil and platelet engraftment occurred in 18 days and 42 days for the patients who received UM171-expanded CBT, respectively, demonstrating good safety. The study indicated a benefit in 12-month overall survival (90%) for a patient population at high risk for TRM (with a high hematopoietic cell transplant comorbidity index) and relapse. The current minimal cell dose criteria limit cord blood selection to only 5% of stored cord blood (for a 70 kg patient), however, with this expansion, they were able to access 47% of stored cord blood, allowing half of these patients to receive a better HLA-matched graft.^58^

##### Omidubicel: Expansion With Nicotinamide

The results of preclinical studies indicate that when UCB-derived hematopoietic progenitor cells are cultured in the presence of nicotinamide and cytokines, a significant increase is observed in the number of primitive CD34+CD38– cells and an improvement in BM homing and engraftment.^59^ Omidubicel is a patient-specific cell product that is derived from a single UCB unit that consists of a CD133+ fraction that has been expanded ex vivo and a non-expanded CD133– a fraction that has been exposed to nicotinamide.^60^

A phase III trial involving 125 patients (adolescents and adults) with hematologic malignancies compared omidubicel with the standard UCBT using a MAC regimen. In the omidubicel arm, the median time to neutrophil engraftment was 12 days, whereas it was 22 days for the control arm. The omidubicel arm demonstrated faster platelet recovery (55% vs. 35%), as well as a lower incidence of bacterial or fungal infection (37% vs. 57%) than the control arm; additionally, there were no significant differences between the 2 groups regarding GvHD or overall survival (70-75%), however, the study was not powered to detect a statistically significant difference in survival.^60^ Based on the results of this study an application for a biologic license for the product was expected to be submitted to the FDA in the first half of 2022. Other methods of ex-vivo expansion are described in Table 3. However, it is imperative to emphasize that, up to date, none of these ex-vivo expansion methods have been adopted as a standard of care in clinical practice.

**Table 3.** Pre-clinical and clinical studies of ex vivo expansion of UCB HSC and HPC.

Reference and year	Expansion method	Scientific rationale	Culture media	Findings
Pecora et al (2001)^114^	Continuous perfusion culture device	Allows expansion of BM nucleated cells and CFU	PIXY321, FLT-3 ligand, EPO, fetal bovine serum, and horse serum.	–All patients had neutrophil and platelet engraftment at 10 and 13 days, respectively.
Jaroscak et al (2003)^115^				–2.4-fold increase in nucleated cells, 82-fold in CFU granulocyte-macrophages, and 0.5-fold in CD34+ cells.
Peled et al (2004)^116^	Copper chelator	Regulates HSC/HPC proliferation and differentiation.	IL-6, SCF, TPO, FLT-3 ligand, and TEPA (copper chelator).	–Significant increments in the numbers of CD34^+^ cells (89-fold), CD34+CD38- (30-fold), and CFU cells (172-fold).
De Lima et al (2008)^117^				–Mean increase of CD34+ cell count was of 6. –Median time to neutrophil and platelet engraftment was 30 and days.
Delaney et al (2010)^118^	Notch signalling pathway	Regulates the development of multiple cell lines and expansion of HSC/HPC.	IL-3, IL-6, TPO, FLT-3 ligand, SCF, and Notch ligand Delta1.	–222-fold increase in CD34^+^ cells. –Significantly enhanced neutrophil engraftment (median time: 16 days).
Fernández-Sánchez et al (2011)^119^			IL-3, IL-6, SCF, FLT-3 Ligand, TPO, GM-CSF, G-CSF, and OP9.	–21-fold increase in D34^+^ CD38^−^ cells.
De lima et al (2012)^55^	Mesenchymal cell (MSC) coculture	Secretes and suppresses endogenous cytokines and forms extracellular matrix networks.	SCF, FLT-3 ligand, TPO, G-CSF, and MSC isolated from BM.	–88% of patients engrafted by day 26. –Median time to neutrophil and platelet engraftment was 15 and 42 days, respectively.
Wagner et al (2015)^120^	StemRegenin-1 (SR-1)	Promotes expansion of CD34+ HPC.	IL-6, SCF, FLT-3 ligand, TPO, and SR-1.	–330-fold increase in CD34+ cells. –All the patients achieved neutrophil engraftment (median time: 15 days).
Faivre et al (2016)^121^	Glycosaminoglycans (GAGs)	Modulate growth factor effects in the hematopoietic niche.	SCF, TPO, FLT3-ligand, G-CSF and OTR4131 (GAG mimetic).	–Cell expansion was not significantly increased. –Mice successfully engrafted.
Bari et al (2018)^122^	Azole-based small molecule (C7)	A type of p38-MAPK inhibitor. Inhibition of p38-MAPK restores hematopoiesis in myelodysplastic syndrome.	SCF, TPO, FLT-3 ligand, IGFBP-2, various cytokines, and C7.	–3.7-fold increase of CD45^+^CD34^+^CD38^–^CD45RA^–^ progenitors. –C7 expands UCB HPC without CD34/CD133-based enrichment.
Horwitz et al (2019)^59^	Nicotidamine (NAM)	Vitamin B3 that inhibits both NAD+-dependent enzymes and the Sirtuin family of histone/protein deacetylases.	SCF, TPO, FLT-3 ligand, G-CSF, and NAM.	–The cumulative incidence of neutrophil engraftment at day 42 was 94%. –The median time to neutrophil recovery was 11.5 days.
Cohen et al (2020)^58^	UM171	Agonist pyrimidoindole derivative that enhances multilineage human blood cell regeneration in immunocompromised mice.	IL3, IL-6, SCF, FLT-3 ligand, and UM171.	–Median time to neutrophil and platelet engraftment was 18 and 42 days, respectively. –No graft failure occurred.

Abbreviations: BM, bone marrow; CFU, colony-forming unit; EPO, erythropoietin; FLT-3, Fms-like tyrosine kinase 3; GAGs, glycosaminoglycans; G-CSF, granulocyte-macrophage colony-stimulating factor; GM-CSF, granulocyte-macrophage colony-stimulating factor; HPC, hematopoietic progenitor cell; HSC, hematopoietic stem cell; IGFBP-2, insulin-like growth factor binding protein-2; IL, Interleukin; MSC, Mesenchymal cell; NAM, Nicotidamine; NK, Natural killer; PIXY321, granulocyte-macrophage colony stimulating factor/interleukin-3 fusion protein; SCF, stem cell factor; SR-1, StemRegenin-1; TEPA, tetraethylenepentamine; TPO, thrombopoietin; UCB, umbilical cord blood.

#### Other Methods

##### Enforced Fucosylation

Although ex vivo expansion of cord blood cells seemed like a promising strategy, it did not address an inherent problem of UCBT, which is the low affinity of a substantial subset of CD34+ cells for the BM microvasculature, leading to a lack of migration and homing of stem cells to their niches. There is evidence to suggest that delayed engraftment may be caused by low levels of fucosylation of cell surface molecules that are responsible for adhering to P- and E-selectins constitutively expressed in the marrow microvasculature, as well as for marrow homing.^61^ To address this deficiency, the MDACC group conducted a first-in-human study in which 22 patients with hematologic malignancies received MAC regimen and 2 cord blood units treated ex vivo with the enzyme fucosyltransferase-VI and guanosine diphosphate fucose to improve the interaction of CD34(+) stem cells and early progenitors with microvessels. The patients were compared with historical control participants who had undergone unmanipulated dUCBT.^8^

In the fucosylation group, the median time to neutrophil and platelet engraftment was 17 and 35 days, respectively, compared with 26 and 45 days in the control group. The cumulative incidence of grades II–IV acute GvHD was 41%, compared with 39% in controls, and the cumulative incidence of chronic GvHD was 5% compared with 23%. Only 45% of patients survived an 8-month follow-up period in this study. In a similar manner to the authors of the ex-vivo mesenchymal cell coculture study, lower survival rates were attributed to the fact that the majority of patients (two-third) had underlying high- or very high-risk diseases at the time of treatment. Several factors have been proposed to explain the accelerated engraftment: fucosylation likely increased the proportion of infused cells that were able to interact with E- and P-selectins, and therefore to be recruited to the bone marrow niches. Moreover, fucosylation of other cells, such as natural killers (NK) and monocytes, facilitated the generation of neutrophils and platelets from HSCs. Based on these findings, ex vivo fucosylation of cord blood cells was deemed a feasible method for improving engraftment efficiency in the context of dUCBT.^8^

##### Intrabone Route

The intrabone (IB) route has been proposed as a method to overcome the limitation of the low stem cell dose of UCB. The injection of UCB HSC into the BM is believed to expose the cells directly to beneficial stimuli provided by the HSC niche.^62^ In this procedure, UCB units are thawed, washed, and re-suspended in albumine–dextran solution. Following analgo-sedation in the operating room, UCB is injected directly into the posterior superior iliac crests of the patient lying on a side, repeating the procedure 4-6 times. Patients are then transferred back to their postoperative rooms.^63^

UCBT (single unit) via IB route was evaluated in a phase II trial with patients with very advanced hematological malignancies, and no serious adverse events were reported relating to the infusion. Furthermore, the cumulative incidence of neutrophil and platelet engraftment at day 90 was 82% and 70%, respectively, and the overall survival at 12 months was approximately 65%. There was no severe acute GvHD and only 1 case of extensive chronic GvHD. CD3 + T cells recovery was faster than with IV infusion.^63^ In pediatric population, the rate of neutrophil engraftment and severe acute GvHD with IB administration were similar to those historically seen with IV administration.^64^ Studies comparing IB and IV infusion have not yet shown clear advantages for IB infusion, and with the additional costs for transplant procedures in the operating room, this route of administration remains experimental.^62-64^

## Umbilical Cord-Blood-Derived Natural Killer (NK) Cells Used in Cancer Immunotherapy

Immunotherapy based on UCB-derived Natural Killer (NK) cells has shown promise in the treatment of cancer in recent years.^65^ The umbilical cord is a rich source of NK cells, a type of large granular lymphocyte of the innate immune system that plays an essential role in anti-cancer immunity.^66,67^ A set of receptors (particularly NKG2A) allows NK cells to target tumor cells non-specifically and independently of the major histocompatibility complex.^66,67^ Genetically engineered NK cells express a chimeric antigen receptor (CAR) construct that consists of an extracellular synthetic receptor for antigen recognition, an extracellular hinge region, a transmembrane domain, and one or more intracellular co-stimulatory domains.^68,69^ Engineered and expanded NK cells provide an additional layer of anti-tumor activity while maintaining their native capacity to recognize tumor cells.^68^ Furthermore, NK cells do not rely on the T cell receptors for cytotoxic killing, thus giving them a more favorable safety profile than their T-cell counterparts, which, in the allogeneic setting, may reduce the risk of GvHD.^68^

CAR-NK cell therapy (a third-generation CAR lentiviral construct containing CD28 and 4-1BB co-stimulatory molecules) showed safety in phases 1 and 2 clinical trials with CD19+ lymphoid neoplasms without developing cytokine release syndrome (CRS) or neurotoxicity.^70^ Nevertheless, the in vivo duration of the therapy is limited. To improve the duration of the product in vivo, a CAR-NK product with constitutive IL-15 cytokine support was developed.^71,72^ After receiving this product, 11 patients with relapsed and refractory (R/R) lymphoid tumors, 8/11 (73%) achieved a clinically significant response, with 7/11 (63%) achieving a complete remission. The safety profile was excellent.^69^

An ongoing phase I clinical trial of an induced pluripotent stem cell (iPSC)-derived NK cell product, FT516, which expresses a novel high-affinity non-cleavable 158V CD16 Fc receptor that has been modified to enhance its binding to tumor-targeting antibodies, was provided to 11 patients with R/R B-non-Hodgkin lymphoma. Among 4 patients who received a dose of more than 90 million cells/dose in combination with rituximab, 3 showed an objective response without signs of CRS, neurotoxicity, or GvHD.^72^ More follow-up data will be required to determine whether the observed responses are durable.

Despite these promising results, CAR-NK has many limitations including the short in vivo duration of the adoptive NK cells, for which novel modalities have been implemented (IL-15 gene transduced into NK cells).^69,73^ As a final point, it has not been determined which conditioning regimen to use, what time point to infuse NK cells, and what the optimal range is for infusion of NK cells, nor whether any allogeneic products should be used.^73,74^ Larger clinical trials need to confirm these findings and assess long-term response duration.

## Future Directions and the use of UCB-Derived Cells in Other Settings

Allogeneic stem cell transplantation patients are at risk for opportunistic infections.^75^ Adenovirus, cytomegalovirus (CMV), and Epstein-Barr virus (EBV) are among the viruses that can be treated using Virus-specific T-cell (VST) therapy, which is safe, effective, and has a long-term persistence in vivo.^76-78^ The umbilical cord provides a source of nave T cells that are capable of recognizing atypical viral epitopes while keeping their functionality.^79^ In 2 clinical trials conducted at 2 separate sites, UCB-derived multivirus-specific T cells (CB-VSTs) targeting EBV, adenovirus, and CMV that were expanded ex-vivo, were used in 14 patients who underwent UCBT: 7 patients as prophylaxis and 7 for treatment.^77^ None of the 7 patients who received CB-VSTs as prophylaxis developed end organ disease from the viruses, and only 1/7 patients receiving CB-VSTs for viral reactivation or infection experienced end organ viral disease (CMV retinitis) in the setting of steroid therapy for GvHD. There was no evidence of delayed engraftment or GvHD. In high-risk patients, CB-VSTs may maintain antiviral immunity and complement antiviral pharmacotherapies to prevent viral infection.^77^

Beyond the clinical domain of oncology, stem cells and mesenchymal stem cells (MSC) derived from UCB units have been investigated for multiple conditions, as shown in Table 4. UCB-derived stem cells have been shown to improve the restoration of limb function following spinal cord injury (SCI) when administered systemically and/or intrathecally.^80-87^ It is believed that stem cells stimulate the synthesis of interleukin 10, glial cell line-derived neurotrophic factor (GDNF), and vascular endothelial growth factor (VEGF), and reduce pro-apoptotic factors such as Fas proteins and caspases.^88,89^ Pre-clinical studies have demonstrated that UCB-MSC improves hippocampal neurogenesis and synaptic activity in mature mice with neurodegenerative diseases, such as Alzheimer’s disease (AD).^90-92^ In humans, the benefits of this therapy have not been replicated although more than a dozen trials are ongoing to determine whether it can be effective in treating AD.^93,94^

**Table 4.** Non-malignant conditions treated with UCB-derived MSC in the most relevant clinical studies.

Condition	Reference and year	Patient population	Methods	Outcomes
Neurological conditions				
Spinal cord injury (SCI)	Yao et al (2013)^80^	50 chronic traumatic SCI patients divided into 2 groups: UCB stem cells plus traditional rehabilitation (*N* = 25); and the control group (traditional rehabilitation, *N* = 25).	IV and IT administration of UCB stem cells plus traditional rehabilitation.	–Improvements in neurological function and somatosensory evoked potential tests. –There were no severe AEs.
	Liu et al (2013)^81^	22 patients with chronic SCI received UCB-MSC.	IT administration of UCB-MSC given 4 times.	–Effective in 60% of patients. –Motor and/or sensory functions, and bowel and bladder control were improved.
	Cheng et al (2014)^82^	34 patients with SCI were randomly divided into 3 groups: the UCB-MSC (*n* = 10); the rehabilitation; and the control groups (*N* = 24 in the last 2 groups).	IT administration of UCB-MSC.	–In the UCB-MSC group, 70% of patients improved in movement, self-care, and muscular tension, compared to 36% in rehabilitation.
	Zhu et al (2016)^83^	28 patients with chronic SCI received UCB-MNC divided in 5 groups in 2 phases: phase I (= 8) and phase II (*N* = 20).	IT administration of UCB-MNC at different dose levels. One group also received corticosteroids, another group received lithium. On phase II: 17 patients received intensive locomotor training.	–At 1 year 75% of phase II patients were able to walk and 60% did not need assistance for bladder or bowel management.
	Deng et al (2020)^84^	40 patients with acute complete SCI were divided in 2 groups: UCB-MSC (*N* = 20); and the control groups (*N* = 20).	IT administration of the UCB-MSC.	–Activity score of daily life scores was increased, as well as bowel and urinary functions. –MRI showed that new nerve fiber connections were formed.
	Yang et al (2021)^85^	102 patients with chronic SCI received UCB-MSC.	IT administration of UCB-MSC monthly for 4 courses.	–Significant increase in motor scores compared to the baseline.
	Zhao et al (2021)^86^	7 patients with SCI were divided in 2 groups: UCB-MSC and rehabilitation treatment (*N* = 4); and the control groups (only rehabilitation, *N* = 3).	IV and IT administration of UCB-MSC were administered.	–Neurological improvement after treatment.
	Albu et al (2021)^87^	10 patients with chronic SCI were randomized to receive WJ-MSC or placebo (crossover was allowed at 6 months).	Intrathecal administration of WJ-MSC. In every patient, 2 infusions were administered (WJ-MSCs first and then placebo infusion or placebo first and then WJ-MSC infusion), separated by 6 months).	–Pinprick sensation improved significantly in dermatomes below the level of injury. –Neither motor function, spasticity, bowel function, nor quality of life were affected.
Alzheimer’s disease (AD)	Jin Kim et al (2015)^93^	9 patients with AD received UCB-MSC.	Percutaneous stereotactic injection of UCB-MSC into the hippocampus.	–After treatment, the rate of cognitive decline was faster than in typical AD. The authors suggested that this was because 7/9 patients were early onset AD, which is known to progress faster than typical late-onset AD. –No serious AEs.
	Jin Kim et al (2021)^94^	9 patients with mild-to-moderate AD received UCB-MSC.	UCB-MSC administration via Ommaya reservoir into the right lateral ventricle. Three injections at 4-week intervals. Patients received 2 dose levels.	–Reduced levels of total and phosphorylated tau, and Aβ42 in the CSF samples after treatment. –All participants experienced fever. –Clinical efficacy of UCB-MSC could not be proven.
Cardiovascular conditions				
Heart failure (HF)	Zhao et al (2015)^96^	59 patients with ischemic and/or dilated cardiomyopathy were divided in 2 groups: UCB-MSC and drug therapy (*N* = 30); and the control groups (*N* = 29) (only drugs).	UCB-MSC was administered via intracoronary injection.	–Alleviation of HF symptoms occurred 24 h after infusion. –Decrease in mortality. –Significant increase in the 6-min walking distance, LVEF, and improvement of NT-proBNP levels after treatment.
	Fang et al (2016)^97^	3 patients with HF caused by ischemic cardiomyopathy received UCB-MSC.	UCB-MSC was administered intravenously.	–Two-third of patients had an increase in LVEF of 65% by the end of the third month. –Significant improvements in the clinical parameters of the NYHA functional class and 6-min walk test. –No severe AEs.
	Bartolucci et al 2017)^95^	30 patients with HF and chronic low LVEF under optimal medical treatment were randomized: UCB-MSC (*N* = 15); and the control groups (*N* = 15).	IV administration of UCB-MSC.	–Improvement in LVEF at 3, 6, and 12 months of follow-up assessed by TTE and cardiac MRI. –Improved NYHA functional class and QoL. –There were no differences in mortality, arrhythmias, or HF.
Myocardial infarction (MI)	Musialek et al (2015)^123^	10 patients with acute MI with successful percutaneous coronary intervention received WJ-MSC.	WJ-MSC was administered by coronary artery injection.	–A transient rise in temperature was the only AE associated with WJ-MSC administration.
	Li et al (2015)^124^	15 patients with chronic total occlusion of the coronary arteries received UCB-MSC.	UCB-MSC was administered into the epicardial coronary artery at three different dose levels based on random allocation.	–Significant reduction in the size of the infarct and a remarkable increase in LVEF. –No AEs were reported during the study period of 24 months.
	Gao et al (2015)^125^	116 patients with ST-elevation MI were randomly assigned: WJ-MSC (*N* = 58); or placebo (*N* = 58).	WJ-MSC was administered via intracoronary injection on days 5-7 after the MI.	–Measurements of myocardial viability and perfusion demonstrated a reduction in the size of the infarct. –The LVEF increased at 18 months.
	Ulus et al (2020)^126^	54 patients with chronic ischemic cardiomyopathy were randomized: UCB-MSC or autologous BM-MNC during CABG surgery (*N* = 38); or to undergo CABG alone (*N* = 16).	The stem cells were administered intramyocardially into 10 areas of the peri-infarct region during CABG surgery.	–As measured by TTE and cardiac MRI, LVEF significantly increased in the UCB-MSC group, but not in the BM-MNC or control groups. –The necrotic areas improved.
Respiratory conditions				
Coronavirus disease 19 (COVID-19)	Meng et al (2020)^98^	18 patients hospitalized with moderate-severe COVID-19 pulmonary disease were divided into 2 groups: UCB-MSC (*N* = 9); and the control groups (*N* = 9).	Three cycles of intravenous infusion of UCB-MSC on days 0, 3, and 6. Both groups received standard COVID-treatment regimens.	–Mechanical ventilation was required in 1/9 patient in the treatment group compared to 4/9 in the control group. –Only 2 patients developing transient facial flushing and fever, and 1 transient hypoxia after the infusion of cells.
	Shu et al (2020)^99^	41 patients with severe COVID-19 were randomly divided into 2 groups: UCB-MSC (*N* = 12); and the control groups (*N* = 29).	IV infusion of UCB-MSC. Both groups received standard COVID-treatment regimens.	–Patients in the UCB-MSC group did not progress to critical illness within 28 days (vs. 4/29 patients in the control group) and had a shorter time to clinical improvement. –CRP, IL-6 levels, lymphocyte count, and lung inflammation seen on scans returned to normal significantly faster.
	Chen et al (2020)^102^	25 patients with severe COVID-19 who had received UCB-MSC were analyzed retrospectively.	UCB-MSC was administered intravenously. All patients received conventional treatment.	–In all patients, recovery was achieved after therapy, and only 3 patients experienced AEs (liver dysfunction, heart failure, and allergic rash).
	Adas et al (2020)^103^	30 patients with moderate–severe COVID-19 were divided into 3 groups: Group 1 (moderate cases, *n* = 10), Group 2 (critical cases, *n* = 10), and Group 3 (critical cases, *n* = 10, treated with WJ-MSC).	WJ-MSC was administered in 3 doses on days 0, 3, and 6. All patients received conventional treatment.	–6/10 patients in Group-2 and 3/10 patients in Group-3 died, while no patients in Group-1 died. –Group-3 had significantly lower serum ferritin, fibrinogen, and CRP levels compared to Group-2.
	Feng et al (2020)^104^	16 patients with severe–critically severe COVID-19 received UCB-MSC in addition to standard of care.	UCB-MSC was given intravenously every other day in 4 rounds.	–Oxygenation index, opacities on radiological images, and lymphocyte counts (CD4+ T cells, CD8+ T cells, and NK cells) improved.
	Hashemian et al (2021)^105^	11 patients diagnosed with COVID-19-related ARDS were divided in two groups: UCB-MSC (*N* = 6); or placental MSC (*N* = 5).	Patients received three intravenous infusions every other day of UCB- MSC or placental MSC.	–In 7/11 patients, dyspnea and SpO_2_ improved within 48-96 h. –Six patients survived and had significant reductions in TNF-* α*, IL-8, and CRP levels. –The CT scans showed remarkable signs of recovery.
	Lanzoni et al (2021)^106^	24 patients with ARDS due to COVID-19 infection were randomized at a 1:1 ratio: UCB-MSC treatment (*N* = 12); and the placebo groups (*N* = 12).	UCB-MSC was administered intravenously at day 0 and 3. Both groups received best standard of care.	–A significant improvement in survival (91% vs. 42%) and recovery time was associated with treatment. –At day 6, UCB-MSC treated subjects showed a significant reduction in inflammatory cytokines. –No serious AEs.
	Shi et al (2021)^107^	101 patients with severe COVID-19 were randomly assigned at a 2:1 ratio: UCB-MSC (*N* = 65); or placebo (*N* = 35).	UCB-MSC was administered intravenously on days 0, 3, and 6 after randomization.	–Increasing distance in the 6-min walk test and improvement in the lung lesions at day 28. –AEs were similar.
	Iglesias et al (2021)^108^	5 patients with COVID-19-associated ARDS that did not improve after 48 h of receiving standard medical management received UCB-MSC.	UCB-MSC was administered intravenously.	–Two patients died at 13 and 15 days after infusion, while 3 survived and were extubated on the nineth day. –There were no AEs.
	Wei et al (2021)^109^	25 patients with severe COVID-19 were included in 2 groups: UCB-MSC plus conventional therapy (*N* = 12); and the control groups (conventional therapy alone) (*N* = 13).	UCB-MSC was administered intravenously within 42 days of admission.	–Oxygenation index and the inflammatory areas seen on the CT scan improved significantly. –Decrease in IgM levels.
	Dilogo et al (2021)^100^	40 critically ill patients with COVID-19 were randomly allocated: UCB-MSC (*N* = 20); and the placebo groups (*N* = 20).	UCB-MSC was administered intravenously on day 8 of ICU admission. Both groups received best standard of care.	–2.5 times higher survival rate. –Those with comorbidities had a 4.5 times higher survival rate. –Significant reduction in IL-6 levels.
	Zhu et al (2021)^101^	58 patients with severe COVID-19 were randomized in a 1:1 ratio into 2 groups: UCB-MSC group (*N* = 29); and the placebo groups (*N* = 29).	UCB-MSC was administered intravenously.	–Shorter hospital stays and a shorter period of symptom remission. –Decreased CRP, proinflammatory cytokines, and increased antibodies specific to SARS-CoV-2. –Regulation of B-cell subsets and increased CD28 expression in T-cells.

Abbreviations: Aβ42, amyloid β-protein 42; AD, Alzheimer’s Disease; AEs, adverse events; ARDS, acute respiratory distress syndrome; BM-MNC, bone marrow-derived mononuclear cells; CABG, coronary arterial bypass grafting; COVID-19, coronavirus disease 19; CRP, C-reactive protein; CSF, cerebrospinal fluid; CT, computed tomography; HF, heart failure; ICU, intensive care unit; IgM, immunoglobulin M; IL, interleukin; IT, intrathecal; IV, intravenous; LVEF, left ventricular ejection fraction; MI, myocardial infarction; MRI, magnetic resonance imaging; MSC, mesenchymal stem cells; NK, natural killer; NYHA, New York Heart Association; NT-proBNP, N-terminal-pro hormone B-type natriuretic peptide; QoL, quality of life; SARS-CoV-2, severe acute respiratory syndrome *coronavirus 2*; SCI, spinal cord injury; TNFα, tumor necrosis factor *α*; TTE, transthoracic echocardiogram; UCB, umbilical cord blood; UCB-MNC, umbilical cord blood-derived mononuclear cell; UCB-MSC, umbilical cord blood-derived mesenchymal stem cells; WJ-MSC, Wharton’s jelly (umbilical cord matrix) mesenchymal stem cells.

In the treatment of patients with heart failure and reduced ejection fraction, the effect of using UCB-MSC was associated with an improvement in left ventricular function and functional status in the treated individuals (Table 4).^95-97^ Infusion of UCB-MSC to patients with severe COVID-19 infection has also been associated with a significant improvement in survival.^98-109^ Despite their promising use in the medical field, the lack of standardized protocols for the isolation, expansion, and cryopreservation of UCB-MSC makes it difficult to compare the results of experimental studies and clinical trials.^110^

Several clinical trials are currently being conducted to assess the efficacy and safety of this therapy. The safety and efficacy of intraspinal UCB stem cells for acute and subacute (NCT03521336, NCT02481441) or chronic spinal cord injuries (NCT03505034, NCT03521323) are being investigated through clinical trials using the cells alone or in combination with locomotor training (NCT03979742). There are several phases 1-2 combined studies evaluating the safety of UCB-MSC administered intravenously (NCT01547689) or via Ommaya reservoirs (NCT04954534, NCT03172117) in patients with AD. For cardiovascular diseases, non-ischemic cardiomyopathy (NCT04325594, NCT04992832), but also ischemic diseases: intravenous and/or intracoronary administration (NCT04340609, NCT05147766, NCT03902067, NCT04056819, NCT02666391, NCT02439541), intracardiac injections (NCT04939077), or collagen scaffolds injected in the infarct region (NCT02635464) are being investigated. Similarly, Wharton’s jelly-derived mesenchymal stromal cells (WJ-MSCs) administered intravenously (NCT04551456), intracoronary (NCT05043610), or using a pericardial matrix colonized with MSC implanted on the ischemic region of patients undergoing surgical revascularization (NCT03798353, NCT04011059). To treat COVID-19-related pneumonia, there are currently dozens of clinical trials using UCB-MSC (NCT05132972, NCT04273646, NCT04615429, NCT05433298, NCT05286255, NCT04371601, NCT05501418) or WJ-MSC (NCT04896853, NCT04313322) (Available at https://clinicaltrials.gov). In summary, the experience of the last 3 decades suggests that UCBT has potential in the treatment of hematologic and non-hematologic disorders.

## Conclusions

Umbilical cord blood continues to be an alternative source of HSCT in patients with hematologic malignancies, BM disorders, congenital immunodeficiencies, and metabolic disorders.^111^ The use of UCBT has many advantages over other sources of HSCs, such as less stringent HLA matching requirements, rapid access, and potentially lower risk of relapse and GvHD.^2,5,24^ Nonetheless, selection of the appropriate cell dose and concerns regarding delayed hematopoietic and immunological recovery have limited its widespread use.

Numerous methods have been developed in recent decades to enhance immune reconstitution and engraftment, including efforts involving mesenchymal progenitor cell expansion, which demonstrated a rapid increase in neutrophil engraftment when compared to historical controls.^55^ The expansion studies have been promising but have been constrained by limited sample sizes and special technology that may be difficult to apply in most of the centers. Other strategies have been explored to improve the use of UCBT, such as fucosylation, systemic reduction of erythropoietin levels by hyperbaric oxygen, and direct intramarrow injection of the UCB cells, with various results.^8,112,113^

The use of UCB has quickly expanded beyond UCBT, for instance using UCB-derived virus-specific T cells for the treatment of adenovirus, cytomegalovirus, and Epstein-Barr virus infections.^78^ Patients with B-cell malignancies have been treated with anti-CD19 chimeric antigen receptor (CAR)-natural killers (NK) cells derived from UCB, with good tolerability and without the requirement for complete HLA matching, thus displacing the need to produce a unique CAR product for each patient.^69^ In parallel, UCB-MSC have been demonstrated to be safe and variably effective for treating a wide range of non-hematologic conditions, including neurodegenerative diseases, traumatic spinal cord injury, heart failure, and COVID-19-associated pneumonia, demonstrating that this therapy may prove beneficial in the future for therapeutic purposes.

## Data Availability

No new data were generated or analyzed in support of this research.
